# Cannabis Use among Cancer Survivors: Use Pattern, Product Type, and Timing of Use

**DOI:** 10.3390/cancers15245822

**Published:** 2023-12-13

**Authors:** Ikponmwosa Osaghae, Onyema Greg Chido-Amajuoyi, Banda A. A. Khalifa, Rajesh Talluri, Sanjay Shete

**Affiliations:** 1 Department of Epidemiology, The University of Texas MD Anderson Cancer Center, Houston, TX 77030, USA; 2 Department of Epidemiology, Johns Hopkins Bloomberg School of Public Health, Baltimore, MD 21205, USA; 3 Department of Biostatistics, The University of Texas MD Anderson Cancer Center, Houston, TX 77030, USA; 4 Department of Data Science, University of Mississippi Medical Center, Jackson, MS 39216, USA; 5 Division of Cancer Prevention and Population Sciences, The University of Texas MD Anderson Cancer Center, Houston, TX 77030, USA

**Keywords:** marijuana, cannabis use, cannabis products, oncology, cancer management, cancer survivors

## Abstract

**Simple Summary:**

There is a need for alternative and effective approaches to manage the cancer-related symptoms experienced by survivors. This cross-sectional study describes the current state of cannabis use and how cancer survivors use cannabis during their cancer management to alleviate their cancer-related symptoms including use pattern, product type, and mode of delivery of cannabis. Nearly half of the survivors had used cannabis at some point in their lifetime. Among survivors using cannabis, about half said their cannabis use had increased since cancer diagnosis. The most common types of cannabis products used were dry-leaf cannabis, cannabidiol (CBD) oil, and cannabis candy. Regarding the method of use, most survivors preferred to inhale or smoke cannabis rather than eat or drink it. The most common inhalational methods were rolled cannabis cigarettes, pipes, water pipes, vaporizers, and e-cigarette devices. The study highlights the importance of educating healthcare providers and survivors about cannabis use during cancer treatment.

**Abstract:**

Despite growing interest in the use of cannabis for the treatment of cancer-related symptoms, there are limited studies that have assessed the use pattern, type, and mode of delivery of cannabis products used by cancer survivors. This study describes the current state of the use pattern, product type, and mode of delivery of cannabis used by cancer survivors. This was a cross-sectional study of cancer survivors from 41 U.S. states who received treatment at the largest NCI-designated comprehensive cancer center. The weighted prevalence of the use patterns, product types, and modes of delivery of cannabis used by cancer survivors was estimated. A total of 1886 cancer survivors were included in the study, with 915 (48% [95% CI: 45–51]) reporting ever using cannabis. Of survivors who had ever used cannabis, 36% (95% CI: 33–40) were current users. Among survivors who reported cannabis use after diagnosis, 40% used cannabis during and after cancer treatment, 35% used cannabis during treatment, and 25% used cannabis after completing their cancer treatment. Additionally, 48% of survivors reported an increase in cannabis use since cancer diagnosis. The commonest types of cannabis products used by cancer survivors were dry leaf cannabis (71%), cannabidiol (CBD) oil (46%), and cannabis candy (40%). Moreover, cancer survivors frequently used baked goods (32%), creams and gels (21%), and tinctures (18%). Furthermore, among ever users, the predominant mode of use was cannabis inhalation/smoking (69%) compared to eating/drinking (59%). More so, the common mode of inhalation/smoking of cannabis products were rolled cannabis cigarettes (79%), pipes (36%), water pipes (34%), vaporizers or vapes (14%), and e-cigarette devices (14%). A substantial number of cancer survivors use cannabis during cancer treatment, with increased use following cancer diagnosis. The forms and modes of delivery of cannabis varied among survivors, with most survivors inhaling or smoking cannabis. There is a need to educate healthcare providers (HCPs) and survivors on current evidence of cannabis use and strengthen cannabis regulatory frameworks to optimize benefits and minimize adverse events from cannabis use during cancer treatment.

## 1. Introduction

With more than two million new cancer cases identified each year, the number of cancer survivors in the United States (U.S.) is rising [1,2]. Cancer survivors often have to deal with symptoms of chronic pain, nausea and vomiting, anxiety, depression, anorexia, insomnia, and weight loss from the disease itself or its treatment [3,4,5,6]. Cannabis or marijuana is a plant containing several cannabinoids, including tetrahydrocannabinol (THC) and cannabidiol (CBD), with some evidence to support cannabis use in oncology to alleviate cancer-related symptoms and improve survivors’ quality of life [7,8,9,10,11]. Amidst conflicting evidence on the effectiveness and safety of medicinal cannabis in managing cancer symptoms, it is the only effective agent for some cancer patients to ameliorate the debilitating symptoms they experience [12,13,14,15]. Consequently, cannabis use commonly becomes an alternative among survivors in managing their cancer and treatment-related symptoms [16].

Moreover, the legal landscape for cannabis use in oncology is rapidly expanding. About thirty-eight state jurisdictions in the U.S. currently have legislation legalizing cannabis use for medicinal purposes [17]. Regardless of state laws legalizing cannabis for medicinal or recreational purposes, cancer survivors often use cannabis without discussing it with their healthcare providers [18,19]. In Texas, where restrictive cannabis laws only allow limited medicinal cannabis use, nearly ninety percent of cancer survivors receiving cancer care are aware of cannabis use in cancer management, with about a third interested in using it to manage their cancer-related symptoms [16,17]. In another study conducted in a state where cannabis is legalized for both medicinal and recreational purposes, a high rate of interest and active use of cannabis has also been noted among cancer survivors [18]. More so, cancer survivors perceive benefits from cannabis use in managing their cancer-related symptoms, including pain, sleep disorder, nausea, stress, and mood disorder, but are not always aware of the potential health risks associated with cannabis use during their cancer management [20]. Despite this growing desire for cannabis use in treating cancer-related symptoms, survivors do not always receive information on the different cannabis products from their healthcare providers [18].

Cannabis is available in several forms and is consumed through different methods, with inconsistencies in the composition, onset of action, and duration of action of the various cannabis products [15,21]. These forms include dry leaf, CBD oil, tinctures, beverages, candy, and creams or gels [18,22]. Also, cancer survivors have different options for consuming cannabis, including inhalation (smoking or vaporizing), topical application, and oral ingestion [18,21]. Inhalation is the prevalent form of cannabis consumption among cancer survivors, increasing the risk of respiratory diseases in this population [23,24,25]. The ratio of THC to CBD varies for different cannabis products, which could affect their effective dosage and indications [15]. As such, the bioavailability, potential side effects, or drug–drug interaction of cannabis with other cancer drugs differs depending on the form or method of consumption of cannabis by survivors [15,26,27,28]. For instance, the peak plasma concentration and onset of action of cannabis when smoked or vaporized is rapid, with a short duration of action. On the other hand, the peak plasma concentration of cannabis, when ingested orally, is much slower but remains elevated for a longer time [28]. In the U.S., some cannabinoids are currently approved by the Food and Drugs Administration (FDA) for medicinal purposes [29]. This includes dronabinol and nabilone, licensed for the treatment of nausea, vomiting, and HIV-associated poor appetite and weight loss, as well as epidiolex, approved for the treatment of rare and severe seizure conditions.

With increasing access to and use of cannabis for treating cancer-related symptoms, it is essential to understand the different product types and modes of delivery of cannabis utilized by cancer survivors in managing their symptoms. More so, the variability in use patterns, forms, and delivery systems of cannabis amidst the potential for adverse events and interaction of cannabis with other cancer medications necessitates insights into how cancer survivors use cannabis after their diagnosis and during active treatment. This understanding could help healthcare providers plan and tailor treatments for cancer survivors to minimize unwanted adverse events and improve survivors’ quality of life. However, limited studies have examined the use pattern, type, and mode of delivery of cannabis products used by cancer survivors. This study aims to describe the use pattern, product type, and mode of delivery of cannabis used by cancer survivors by using data from the largest comprehensive cancer center in the U.S.

## 2. Methods

### 2.1. Data Source and Study Population

This was a cross-sectional study based on data collected between November 2021 and October 2022 among cancer patients attending The University of Texas MD Anderson Cancer Center (MDACC). MDACC is the largest National Cancer Institute (NCI)-designated comprehensive cancer center in the U.S. with a patient population from all U.S. states. This study was based on a survey aimed at learning about cannabis/marijuana use among cancer patients and survivors to better understand the needs of cancer patients. The inclusion criteria were adult (≥18 years) cancer patients and survivors actively receiving treatment at MDACC during the study or those who had finished treatment within the previous five years. All those who met the inclusion criteria were invited to complete an anonymous online survey in English or Spanish. Due to the anonymous nature of the survey, follow-up reminders and compensations were not sent to participants. The questionnaire used in the study was modified from previously published and validated instruments [18,30,31]. The sampling of participants was stratified based on their sex, race, and active/inactive cancer treatment status. Non-Hispanic Blacks were purposefully oversampled to lower the variance of statistical measures in this population. All participants provided informed consent to take part in the study. MDACC Ethical Review Board provided ethical approval. The study is reported following the Strengthening the Reporting of Observational Studies in Epidemiology (STROBE) guidelines.

### 2.2. Measures

#### 2.2.1. Use Pattern of Cannabis among Cancer Survivors

Multiple questions were used to assess the use pattern of cannabis among cancer survivors. First, survivors were asked, “Have you ever used cannabis/marijuana?” All those who responded “Yes” were classified as “Ever users”, while those who responded “No” were classified as “Never users”. To assess current cannabis use, all ever users were asked, “Are you currently using cannabis/marijuana?” Those who responded “Yes” were classified as current users, while those who responded “No” were classified as former users. Additionally, we assessed the pattern of cannabis use since cancer diagnosis using the survey question, “After your cancer diagnosis, which of the following best describes your cannabis/marijuana use?” Possible responses were “Used only during cancer treatment”, “Used during and after cancer treatment”, and “Used only after completing cancer treatment”. Also, to assess change in cannabis use since cancer diagnosis, current users and those who started using cannabis before their cancer diagnosis were asked, “Since your cancer diagnosis, has your cannabis/marijuana use changed?” Possible responses were “Increased”, “Not changed”, or “Decreased”.

#### 2.2.2. Type of Cannabis Product Used by Cancer Survivors

All ever users were asked to select all that applied to the question “Which cannabis/marijuana products have you used?” The types of cannabis products assessed were “CBD oil”, “Pills”, “Tinctures”, “Concentrates”, “Dry leaf cannabis/marijuana”, “Creams and gels”, “Baked goods”, “Candy”, “Beverages”, and “Others”.

#### 2.2.3. Method or Mode of Delivery of Cannabis Used by Cancer Survivors

Ever users of cannabis were asked if they “inhaled or smoked” cannabis or if they “ate or drank” cannabis. Survivors who selected that they “inhaled or smoked” cannabis and those who chose they “ate or drank” cannabis, were further asked about the number of days in a week they used either method. Additionally, cancer survivors who were ever users of cannabis and who reported they “inhaled or smoked” cannabis were asked to select all that applied to the question, “When you inhale(d) or smoke(d) cannabis/marijuana, what method do/did you use?” The following methods were assessed: rolled cannabis/marijuana cigarettes (also known as “joint”, “blunt”, or “spliff”), water pipe (also known as “bong”), vaporizer (“vape”), pipe, e-cigarette device (via a cannabis/marijuana cartridge), and Others.

### 2.3. Data Analysis

All analyses were weighted to consider the complex sampling methodology utilized in the survey and data collection. Precisely, the weights were adjusted for participants’ non-response and sampling stratification based on survivors’ sex, race, and treatment status. To create the base sampling weights, we used the inverse of the sampling probability by dividing the number of sampled individuals in each stratum (sex, race, and treatment status) by the total number of individuals in that stratum within the entire population. Next, we accounted for non-response by fitting a response propensity model to the survey data. Finally, weights were redistributed based on the response propensity from the non-responders to responders. We estimated the weighted prevalence of use patterns, product types, and modes of delivery of cannabis used by cancer survivors with corresponding 95% confidence intervals. A two-sided *p*-value ≤ 0.05 was considered statistically significant. Statistical analyses were conducted using R version 4.2.1.

## 3. Results

A total of 1886 cancer survivors (11.7% response rate) from 41 U.S. states completed the survey. Of the total study population, 47.7% were over 65 years, 36.9% were between 50 and 64 years, and 15.4% were under 50 years. The majority of respondents were female (56.3%). In addition, 84.5% of the population was non-Hispanic White, 3.0% were non-Hispanic Black, 8.1% were Hispanic, and 4.5% were non-Hispanic Other.

### 3.1. Use Pattern of Cannabis among Cancer Survivors

Of the 1886 survivors included in the study, 915 (47.8% [95% CI: 45.0–50.7]) were ever users of cannabis, while 971 (52.2% [95% CI: 49.3–55.0]) were never users of cannabis (Table 1). Among those who were ever users of cannabis, 36.3% (95% CI: 32.5–40.3) were current users, and 63.7% (95% CI: 59.7–67.5) were former users. In addition, of the 298 survivors who reported cannabis use after diagnosis, 39.8% used cannabis during and after cancer treatment, 35.3% used cannabis during treatment, and 24.8% used cannabis after completing their cancer treatment. Furthermore, since cancer diagnosis, 48.2% of survivors reported an increase, 14.2% reported a decrease, and 37.6% reported no change in cannabis use (Table 1).

### 3.2. Type of Cannabis Product Used by Cancer Survivors

**Figure 1:**
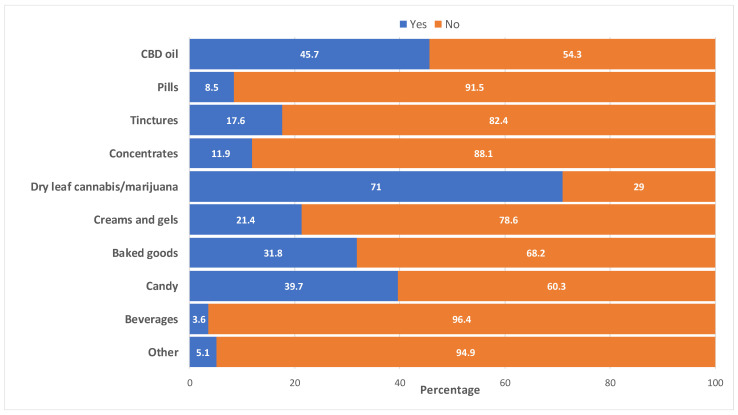
Type of cannabis products used by cancer survivors (*n* = 913 ^a^). ^a^ Ever users of cannabis who responded to the survey question, “Which cannabis/marijuana products have you used? (Check all that apply)”. Two (2) participants did not respond to this question and were missing observation.

As shown in Figure 1, the commonest types of cannabis products used by cancer survivors were dry leaf cannabis (71.0%), CBD oil (45.7%), and cannabis candy (39.7%). Cancer survivors also reported they used baked goods (31.8%), creams and gels (21.4%), and tinctures (17.6%). Other forms of cannabis products reported were concentrates (11.9%), pills (8.5%), and beverages (3.6%) (Figure 1).

### 3.3. Method or Mode of Delivery of Cannabis Used by Cancer Survivors

Among cancer survivors who were ever-users of cannabis, 648 (68.9%) inhaled or smoked cannabis, while 522 (59.2%) of ever-users ate or drank cannabis (Table 2). Of those who inhaled or smoked cannabis, 20.9% inhaled or smoked cannabis daily, while 42.3% inhaled or smoked cannabis less than a day per week. Similarly, of those who ate or drank cannabis, 25.7% ate or drank cannabis daily, while 43.2% ate or drank cannabis less than a day per week (Table 2).

**Figure 2:**
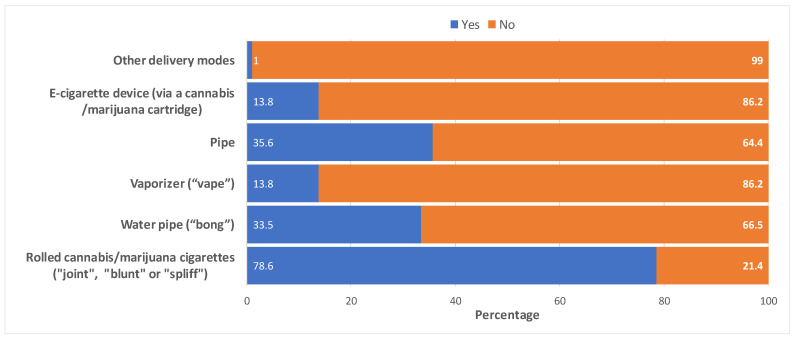
Methods of inhalation or smoking of cannabis among cancer survivors (*n* = 511 ^a^). ^a^ Ever users who inhale or smoke cannabis and who responded to the survey question, “When you inhale or smoke cannabis/marijuana, what method do you use? (Check all that apply)”.

From Figure 2, the commonest mode of inhalation or smoking of cannabis reported by cancer survivors was rolled cannabis cigarettes, also referred to as “joint”, “blunt”, or “spliff” (78.6%). Other common modes of delivery of cannabis were pipes (35.6%) and water pipes, also known as “bong” (33.5%). Less common modes of delivery of cannabis products reported included vaporizers or vapes (13.8%) and e-cigarette devices (13.8%) (Figure 2).

## 4. Discussion

In this study conducted among cancer survivors treated at a leading NCI-designated comprehensive cancer center, one in two survivors used cannabis at some point in their lives, with about half of them stating increased usage after their cancer diagnosis. Dry-leaf cannabis, CBD oil, and cannabis candy were reported as the most prevalent types of cannabis products utilized by survivors. In addition, smoking or inhalation was the preferred mode of using cannabis products. The common methods of inhaling or smoking cannabis products were rolled cannabis cigarettes, pipes, water pipes, vaporizers, and e-cigarette devices.

This study reveals a substantial prevalence of cannabis use among cancer survivors. Nearly half of the participants reported having used cannabis at some point, aligning with findings from other NCI-designated cancer centers that indicate a high prevalence of cannabis use among cancer patients [18,32]. However, results from this study diverge from other population-based studies that typically report lower cannabis usage rates among cancer survivors [23,33,34,35]. This discrepancy may be due to the underrepresentation of cancer survivors in population-based studies, underscoring the strength of this study, which included survivors from forty-one U.S. states. Moreover, this study offers unprecedented insights into the timing of cannabis use within the cancer treatment continuum. Most survivors who initiated cannabis use post-diagnosis did so during their treatment phase. Similarly, regarding changes in cannabis use since cancer diagnosis, approximately half of the survivors (48%) reported increased cannabis use following their diagnosis. This suggests that cancer survivors often turn to cannabis to cope with their diagnosis or manage treatment-related symptoms, corroborating the findings of other studies [18,36,37]. These findings resonate with reports of increased awareness and interest in cannabis use for cancer management among cancer survivors [16]. Survivors from several studies have reported benefits from cannabis use in managing their cancer-related symptoms [7,10]. Consequently, it is vital for the regulatory framework on cannabis use in cancer management to mirror present realities and evidence.

Furthermore, the forms of cannabis and modes of use varied, but the commonest type of cannabis product used by cancer survivors was dry-leaf cannabis, with the predominant mode of use being inhalation or smoking. Correspondingly, most cancer survivors reported that they rolled cannabis cigarettes into joints, blunts, or spliffs and smoked them, while this was closely followed by the use of pipes and water pipes. Other reported forms included CBD oil, cannabis candy, baked goods, creams and gels, tinctures, concentrates, pills, and beverages. This finding aligns with other studies documenting a wide array of product forms and modes of use by cancer patients/survivors [18,19]. The diversity of product choices underscores the range of options available to cancer survivors for cannabis-based symptom management. These findings further highlight the need for healthcare providers to be knowledgeable about these different products and their potential effects.

The findings from this study have several implications. From a regulatory standpoint, cannabis use is widespread among cancer patients; therefore, regulatory guidance is even more critical at this time. As cannabis becomes more accessible for medicinal and recreational use, it is important to strengthen the regulatory framework for its use to minimize the untoward effects of cannabis use in cancer management. While traditional methods of use, such as dried leaf and rolled cannabis, remain popular, this study documents that cancer survivors who use cannabis may integrate novel tobacco products such as vaporizers or vapes and e-cigarette devices. These tools may increase the potential for dual/poly tobacco product use, which further exposes cancer survivors to additional health risks, including developing a second cancer. Moreover, other forms of cannabis, like dried leaves and edibles, may be adulterated with illicit substances, such as opioids, particularly if sourced from non-traditional channels. This underscores the need for the enhanced regulation of cannabis products and their usage. Healthcare providers have crucial roles to play in educating their patients about current evidence on cannabis use, including its indications, potential side effects, and potential interactions with other cancer medications. Such education is important in empowering patients to make informed decisions about using cannabis in cancer management. Moreover, as the body of evidence on cannabis use in cancer management continues to evolve amidst variations in the legal landscape of cannabis across different U.S. states, healthcare providers should continue to receive training on the benefits and risks of cannabis in cancer management. This will enhance their self-efficacy in discussing with and guiding cancer survivors in making informed health decisions regarding their cancer management. 

It is important to acknowledge some limitations of this study. First, the data relied on self-reported information, which may introduce recall bias. Additionally, despite being one of the largest studies on cannabis use in cancer management among cancer survivors, the overall response rate was low. The response rate reported could be attributed to the anonymous nature of the study, intended to reduce information and social desirability bias. More so, the lack of follow-up reminders or compensation for participants may have contributed to the low response rate. However, this study accounted for non-response and potential selection bias by using survey weights to account for non-response in all analyses. Findings from this study could be generalized to other states and settings with similar patient demographics as this study.

This study provides valuable insights into the prevalence, types of cannabis products used, and delivery methods among cancer survivors. The findings suggest that a significant proportion of cancer survivors engage in cannabis use, with a variety of products and routes of administration being utilized. There is an urgent need to strengthen the current regulatory framework on cannabis use in cancer management to optimize benefits and minimize adverse events from cannabis use in managing cancer-related symptoms. Further research is warranted to explore the motivations, efficacy, safety, and long-term outcomes associated with cannabis use among this population, ultimately guiding healthcare professionals in providing informed support and care for cancer survivors.

## 5. Conclusions

Many cancer survivors use cannabis as a palliative while undergoing cancer treatment, and this usage tends to rise following cancer diagnosis. The forms and modes of delivery of cannabis used during cancer management varied among survivors, with most survivors preferring to inhale or smoke medicinal cannabis.

## Figures and Tables

**Table 1:** Pattern of cannabis use among cancer survivors.

Variable	*n* (Wt_*n*)	^a^ Prevalence [95% CI]
Cannabis use (*n* = 1886)		
Never user	971 (34,783)	52.2 [49.3–55.0]
Ever user	915 (31,901)	47.8 [45.0–50.7]
Ever user of cannabis (*n* = 915)		
Former user	579 (20,318)	63.7 [59.7–67.5]
Current user	336 (11,582)	36.3 [32.5–40.3]
Patterns of cannabis use after diagnosis (*n* = 298 ^b^)		
Used during cancer treatment	134 (3840)	35.3 [28.9–42.3]
Used during and after cancer treatment	117 (4330)	39.8 [33.0–47.0]
Used after completing cancer treatment	47 (2701)	24.8 [18.7–32.2]
Change in cannabis use since diagnosis (*n* = 176 ^c^)		
Increased	82 (2801)	48.2 [39.0–57.4]
Not changed	65 (2185)	37.6 [29.2–46.8]
Decreased	29 (827)	14.2 [9.0–21.9]

*n* = Raw number; Wt_*n* = Weighted number; CI = Confidence Interval; ^a^ Prevalence = Weighted prevalence; ^b^ Survivors who started cannabis after cancer diagnosis and were either currently undergoing treatment, finished treatment but undergoing follow-up, or completed treatment and follow-up; ^c^ Survivors who started cannabis before cancer diagnosis and were currently using cannabis.

**Table 2:** Method and frequency of cannabis use among cancer survivors (*n* = 915 ^a^).

Variable	*n* (Wt_*n*)	^b^ Prevalence [95% CI]
Cannabis inhaled or smoked		
No	267 (9919)	31.1 [27.4–35.1]
Yes	648 (21,982)	68.9 [64.9–72.6]
Frequency of smoking or inhalation of cannabis		
<1 day per week	258 (9329)	42.7 [38.0–47.6]
1 day per week	52 (1885)	8.6 [6.3–11.8]
2 days per week	70 (2395)	11.0 [8.3–14.3]
3 days per week	56 (1591)	7.3 [5.3–10.0]
4 days per week	27 (765)	3.5 [2.2–5.5]
5 days per week	29 (975)	4.5 [3.0–6.7]
6 days per week	10 (319)	1.5 [0.7–3.0]
7 days per week (daily)	140 (4564)	20.9 [17.4–25.0]
Cannabis ate or drank		
No	393 (13,021)	40.8 [36.9–44.8]
Yes	522 (18,880)	59.2 [55.2–63.1]
Frequency of eating or drinking cannabis		
<1 day per week	208 (8055)	43.2 [37.9–48.7]
1 day per week	43 (1320)	7.1 [ 4.9–10.2]
2 days per week	54 (1536)	8.2 [5.9–11.4]
3 days per week	44 (1479)	7.9 [5.5–11.4]
4 days per week	25 (758)	4.1 [2.4–6.8]
5 days per week	16 (578)	3.1 [1.7–5.7]
6 days per week	8 (115)	0.6 [0.3–1.4]
7 days per week (daily)	117 (4796)	25.7 [21.2–30.9]

*n* = Raw number; Wt_*n* = Weighted number; CI = Confidence Interval; ^a^ Survivors who reported ever using cannabis; ^b^ Prevalence = Weighted prevalence.

## Data Availability

Data are available from the corresponding author upon request.
